# Rigidez Arterial e Previsão de Doença Renal Crônica

**DOI:** 10.36660/abc.20230779

**Published:** 2024-02-16

**Affiliations:** 1 Universidade Federal de Goiás Liga de Hipertensão Arterial Goiânia GO Brasil Universidade Federal de Goiás - Liga de Hipertensão Arterial, Goiânia, GO – Brasil; 2 Universidade Federal de Goiás Faculdade de Medicina Goiânia GO Brasil Faculdade de Medicina - Universidade Federal de Goiás (UFG), Goiânia, GO – Brasil

**Keywords:** Rigidez Arterial, Insuficiência Renal Crônica, Hipertensão, Endotélio Vascular, Fatores de Risco

Os sistemas renal e cardiovascular têm uma ligação funcional e essa interação tem recebido atenção crescente. A rigidez arterial está frequentemente associada à doença renal crônica (DRC), e ambas as condições podem agravar-se mutuamente, gerando e aumentando o risco de eventos cardiovasculares e mortalidade.^1^ Existe um caminho de mão dupla entre rigidez arterial e disfunção renal. O aumento da rigidez arterial e o endotélio disfuncional ativam citocinas que favorecem eventos trombóticos, e células dendríticas e linfócitos potencializam a síntese de citocinas pró-aterogênicas.^2^ A inflamação vascular amplificada pela DRC estimula o enrijecimento dos vasos pela proliferação das células musculares lisas vasculares e pela fibrose.^2^

Dessa maneira, para tentar responder ao dilema sobre o que veio primeiro, o estudo de Cândido et al.^3^ investigou a relação entre o enrijecimento arterial medido pela velocidade da onda de pulso carotídeo-femoral (VOPcf) e a DRC. Esse estudo longitudinal acompanhou 11.647 participantes do estudo ELSA-Brasil durante 4 anos. Os pesquisadores concluíram que uma VOPcf mais alta aumentou as chances de DRC em 42% e sugeriram que entre participantes normotensos e não diabéticos esse efeito era ainda mais pronunciado, indicando que a rigidez arterial poderia ser um fator de risco significativo para a DRC, independentemente dessas condições.

O aumento da VOP e da pressão arterial sistólica central têm sido identificados como preditores independentes de futuros eventos cardiovasculares e lesões de órgãos-alvo, como hipertrofia ventricular esquerda e aumento da microalbuminúria, e representam os limites entre fatores de risco cardiovascular e eventos cardiovasculares.^4^ O Systolic Blood Pressure Intervention Trial (SPRINT) concluiu que a pressão arterial sistólica < 120mmHg não foi capaz de reduzir a progressão da DRC, mas reduziu o risco cardiovascular e a mortalidade em adultos sem diabetes,^5^ mostrando a natureza multifatorial da progressão da DRC. O estudo CRIC mostrou que a VOP estava altamente associada à doença cardiovascular prevalente e essa associação era independente da pressão arterial sistólica.^6^ O Framingham Heart Study avaliou 7.283 indivíduos por 15 anos e verificou que um incremento do desvio padrão na VOPcf estava associado a um risco aumentado de DRC (1,19, intervalo de confiança de 95% 1,05 a 1,34),^7^ mostrando um resultado semelhante a outros estudos longitudinais.^8,9^

Evidências atuais indicam enrijecimento arterial acelerado em crianças com DRC, mas não há explicação exata para seus mecanismos e consequências.^10^ Existe uma relação alinhada com o aumento do risco cardiovascular e anormalidades estruturais do ventrículo esquerdo.^11,12^ Existem múltiplas razões para esse processo, incluindo pressão arterial elevada, ativação do sistema renina-angiotensina, alterações na matriz extracelular vascular, produtos finais de glicação avançada, estresse oxidativo, disfunção endotelial, calcificação vascular e distúrbios metabólicos ósseos e minerais.^11,12^ Claramente, a DRC e o aumento da VOP estão intimamente interligados e podem influenciar-se mutuamente de maneira bidirecional.^13^

Portanto, a relação recíproca entre o aumento da rigidez arterial e a DRC ressalta a intrincada interação entre a saúde renal e cardiovascular. O monitoramento da VOP, juntamente com outros fatores de risco cardiovasculares clássicos, pode ser importante na identificação de indivíduos com maior risco de desenvolver DRC e outras condições relacionadas. A detecção precoce e o manejo da rigidez arterial são importantes na prevenção da progressão das doenças cardiovasculares e suas complicações. Esses marcadores e preditores podem ajudar os profissionais de saúde a avaliar e gerenciar a saúde cardiovascular de forma eficaz.
