# Supplementary material for: Rigidez Arterial e Previsão de Doença Renal Crônica
Source: Arq Bras Cardiol. 2024 Feb 16;120(12):e20230779. [Article in Portuguese] doi: 10.36660/abc.20230779 (PMC11021035; doi:10.36660/abc.20230779)
Supplement: Translation

# Translation: Arterial Stiffness and Chronic Kidney Disease Prediction

**Affiliations:** 1 Goiânia GO Brazil Universidade Federal de Goiás - Liga de Hipertensão Arterial, Goiânia, GO – Brazil; 2 Goiânia GO Brazil Faculdade de Medicina - Universidade Federal de Goiás (UFG), Goiânia, GO – Brazil

**Keywords:** Arterial Stiffness, Kidney Diseases, Hypertension, Endothelium, Risk Factors

The kidney and the cardiovascular system have a functional connection, and this interaction has received increasing attention. Arterial stiffness is often associated with chronic kidney disease (CKD), and both conditions can aggravate each other, generating and increasing the risk of cardiovascular events and mortality.^1^ There is a two-way path between arterial stiffness and renal dysfunction. Increased arterial stiffness and the dysfunctional endothelium activate cytokines that favors thrombotic events, and dendritic cells and lymphocytes enhance the synthesis of pro-atherogenic cytokines.^2^ The vascular inflammation amplified by CKD stimulates vessel stiffening by proliferation of the vascular smooth muscle cells and fibrosis.^2^

Thus, to attempt to answer the dilemma of which came first, the study by Cândido et al.^3^ investigated the relationship between arterial stiffening by carotid-femoral pulse wave velocity (cfPWV) and CKD. This longitudinal study followed up 11,647 participants of the ELSA-Brasil study for 4 years. The researchers concluded that higher cfPWV increased the chances of CKD in 42% and suggested that among normotensive, non-diabetic participants, this effect was even more pronounced, indicating that arterial stiffness could be a significant risk factor for CKD independent of these conditions.

Increased PWV and central systolic blood pressure have been identified as independent predictors of future cardiovascular events and target organ lesions, such as left ventricular hypertrophy and increased microalbuminuria, and they represent the limits between cardiovascular risk factors and cardiovascular events.^4^ The Systolic Blood Pressure Intervention Trial (SPRINT) concluded that systolic blood pressure < 120mmHg was not able to reduce CKD progression, but it reduced cardiovascular risk and mortality in adults without diabetes,^5^ showing the multifactorial nature of CKD progression. The CRIC study showed that PWV was highly associated with prevalent cardiovascular disease and this association was independent of systolic blood pressure.^6^ The Framingham Heart Study evaluated 7,283 subjects for 15 years, found that a standard deviation increment in cfPWV was associated with increased risk of CKD (1.19, 95% confidence interval 1.05 to 1.34),^7^ showing a similar result to other longitudinal studies.^8,9^

Current evidence indicates accelerated arterial stiffening in children with CKD, but there is no exact explanation for its mechanisms and consequences.^10^ There is a relationship aligned with increasing cardiovascular risk and left ventricular structural abnormalities.^11,12^ There are multiple reasons for this process, including high blood pressure, activation of the renin-angiotensin system, alterations in the vascular extracellular matrix, advanced glycation end products, oxidative stress, endothelial dysfunction, vascular calcification, and metabolic bone and mineral disorders.^11,12^ Clearly, CKD and increased PWV are closely interconnected and can influence each other in a bidirectional manner.^13^

Therefore, the reciprocal relationship between increased arterial stiffness and CKD underscores the intricate interplay among renal and cardiovascular health. Monitoring PWV, along with other cardiovascular classical risk factors, can be important in identifying individuals at higher risk of developing CKD and other related conditions. Early detection and management of arterial stiffness are significant in preventing the progression of cardiovascular diseases and their complications. These markers and predictors can help healthcare professionals evaluate and manage cardiovascular health effectively.
